# Characterization of Extended-Spectrum β-Lactamase-Producing and AmpC β-Lactamase-Producing *Enterobacterales* Isolated from Companion Animals in Korea

**DOI:** 10.3390/antibiotics10030249

**Published:** 2021-03-03

**Authors:** Se Ra Shin, Seong Mi Noh, Woo Kyung Jung, Sook Shin, Young Kyung Park, Dong Chan Moon, Suk-Kyung Lim, Yong Ho Park, Kun Taek Park

**Affiliations:** 1 Department of Pathobiology and Preventive Medicine, College of Veterinary Medicine and Research Institute for Veterinary Science, Seoul National University, Seoul 08826, Korea; shinsr4027@snu.ac.kr (S.R.S.); miss2977@snu.ac.kr (S.M.N.); baram8388@snu.ac.kr (S.S.); pyk1004@snu.ac.kr (Y.K.P.); yhp@snu.ac.kr (Y.H.P.); 2 BK21 PLUS Creative Veterinary Research Center, Seoul National University, Seoul 08826, Korea; 3 Bacterial Disease Division, Animal and Plant Quarantine Agency, Gimcheon 39660, Korea; ansehdcks@korea.kr (D.C.M.); imsk0049@korea.kr (S.-K.L.); 4 Department of Biotechnology, Inje University, Gimhae 50834, Korea

**Keywords:** extended-spectrum cephalosporins, extended-spectrum β-lactamases, AmpC β-lactamases, antimicrobial resistance, Gram-negative bacteria, companion animal

## Abstract

The emergence of extended-spectrum cephalosporin (ESC)-resistant Gram-negative bacteria is of great concern in both human and veterinary medicine. The aim of this study was to investigate ESC-resistant bacterial isolates from companion animals in South Korea between 2017 and 2019. Isolates with ESC resistance genes, which were identified by PCR, were assessed for genetic relatedness by multi-locus sequence typing (MLST) and pulsed-field gel electrophoresis (PFGE). In total, 91 ESC-resistant *Escherichia coli, Klebsiella* spp., *Serratia* spp., and *Enterobacter cloacae* isolates harbored the *bla*_TEM_ gene. Among other ESC resistance genes, *bla*_CTX-M-15_, *bla*_CIT_, and *bla*_CTX-M-55_ were predominantly detected in *E. coli* isolates, whereas *bla*_SHV_ and *bla*_DHA_ were more frequently detected in *Klebsiella pneumoniae* isolates. In addition, all *bla*_EBC_-positive isolates were classified as *E. cloacae*. From the MLST results, *bla*_CTX-M-9_-carrying ST131, *bla*_CIT_-carrying ST405, and *bla*_CTX-M-1_-carrying ST3285 strains were dominant among *E. coli* isolates. ST273 and ST275 strains harboring *bla*_SHV_ were frequently detected in *K. pneumoniae* isolates. Various sequence types were obtained in *E. cloacae* and *Klebsiella oxytoca* isolates. All isolates demonstrated unique PFGE profiles (<57–98% similarity) and were unlikely to be derived from a single clone. The present study reveals the presence and wide genetic distribution of ESC-resistant bacterial species in South Korean companion animals.

## 1. Introduction

The emergence and prevalence of β-lactam resistance in Gram-negative bacteria has increased consistently over the past few decades [1,2]. Resistance to β-lactams is mostly caused by bacterially produced β-lactamases that hydrolyze and inactivate extended-spectrum cephalosporins (ESCs), such as third and fourth generation cephalosporins [1]. ESC resistance is mainly caused by the expression of extended-spectrum β-lactamase (ESBL) and AmpC β-lactamase (AmpC) genes that are normally encoded on mobile genetic elements, mostly plasmids [1].

The first ESBLs have evolved from the native β-lactamases TEM and SHV via genetic mutations [3]. CTX-M β-lactamases, a new group of plasmid-mediated ESBLs, were first reported in Japan in 1986 [4]. However, since 2000, CTX-M β-lactamases have increasingly been reported in both human and animal populations and are now the dominant type of ESBL, replacing classical TEM- and SHV-type ESBLs in most areas of the world [5]. Currently, there are >120 different CTX-M β-lactamases that are clustered into five groups (CTX-M-1, 2, 8, 9, and 25) [6]. Among CTX-M-type enzymes, the presence of CTX-M-15 and CTX-M-14 has increasingly been reported in most areas of the world, including South Korea [5,7].

Since the detection of CMY-1, the first reported AmpC type β-lactamases, in 1989 [6], various types of AmpCs have been identified in clinical isolates of Enterobacterales around the world [8]. Among AmpCs, CIT- and DHA-type enzymes are the most prevalent [2]. Especially, DHA-producing *Klebsiella* spp. isolates and CIT-producing *Escherichia coli* isolates have been repeatedly reported for Enterobacterales in South Korea [2,9]. Despite many studies on ESBL- and AmpC-producing bacteria from human or livestock isolates, studies concerning antimicrobial resistance (AMR) bacteria associated with companion animals are lacking, especially with respect to *Serratia* spp. and *Enterobacter* spp. [10,11,12].

The popularity of companion animals in South Korea has been growing, which provides a potential reservoir of AMR bacteria, as pets are closely associated with humans, living in their homes and near their food [9]. Thus, the importance of profiling AMR bacteria was emphasized in the “One Health” initiative, which integrates veterinary medicine, human health, animal-production systems, and the environment [13]. Systematic control and prevention, through implementation of a national AMR surveillance program, is greatly needed and should be applied in both human and veterinary clinical medicine. The goal of the current study was to investigate AMR among bacterial isolates belonging to Enterobacterales in companion animals within the province of South Korea, with an emphasis on ESC resistance genes in *E. coli*, *Klebsiella* spp., *Serratia* spp., and *Enterobacter cloacae*.

## 2. Results

### 2.1. ESC Resistance Gene Detection

Among the 91 ESC-resistant Enterobacterales isolates analyzed in this study, all of the isolates harbored the *bla*_TEM_ gene. *bla*_CTX-M_ (*n* = 42, 82.4%) was abundantly detected in *E. coli* isolates, whereas *bla*_SHV_ (*n* = 16, 94.1%) was mainly detected in *Klebsiella pneumoniae* isolates (Table 1). None of the *Serratia* spp. and *E. cloacae* isolates, except one *S. marcescens* isolate from *Serratia* spp. that harbored *bla*_SHV,_ were positive for *bla*_SHV_ or *bla*_CTX-M_ (Table 1). Among 55 *bla*_CTX-M_-positive Enterobacterales isolates, *bla*_CTX-M-15_ (*n* = 23, 41.8%) and *bla*_CTX-M-55_ (*n* = 12, 21.8%) were the most commonly detected, followed by *bla*_CTX-M-14_ (*n* = 7, 12.7%) (Table 2). Enterobacterales isolates carrying genes of *bla*_CTX-M-3_, *bla*_CTX-M-61_, *bla*_CTX-M-27_, and *bla*_CTX-M-65_ were also identified (Table 2). *bla*_CTX-M-2_, *bla*_CTX-M-8_, and *bla*_CTX-M-25_ gene clusters were not detected in any isolates. Among *bla*_AmpC_ genes, *bla*_CIT_ was prevalent in *E. coli* isolates, *bla*_DHA_ was predominantly detected in *Klebsiella* spp. isolates, and *bla*_EBC_ was common in *E. cloacae* isolates (Table 3). No isolates carrying genes for *bla*_MOX_ or *bla*_ACC_ were detected.

### 2.2. Multi-Locus Sequence Typing (MLST)

Various sequence types (STs) were revealed among *E. coli*, *K. pneumoniae*, *K. oxytoca*, and *E. cloacae* isolates. STs of *Serratia* spp. isolates were not defined due to the lack of an MLST scheme for this genus. Among *E. coli* isolates, five ST131 and two ST3285 strains were detected from hospital-admitted dogs, with one of each of the following STs: ST372, ST457, ST648, ST1981, ST2179, ST2505, ST4616, ST5150, ST5667, ST8451, ST8885, ST8908, ST10207, ST10220, and ST11000. Accordingly, five ST405, three ST354, two each of ST3285, ST410, ST448, and ST457, and one each of ST68, ST38, ST648, ST1193, ST2541, ST7644, and ST10459 strains were detected in stray dogs. Among six *E. coli* isolates from hospital-admitted cats, two ST131, two ST156, and one each of ST1262 and ST6105 strains were obtained. In a comprehensive analysis of 19 *Klebsiella* spp. isolates from hospital-admitted dogs, one ST285, eight ST275, and six ST273 stains were identified among *K. pneumoniae* isolates, whereas one each of ST34, ST145, ST273, and ST293 strains were identified among *K. oxytoca* isolates. All *Klebsiella* spp. isolates from hospital-admitted and stray cats were identified as ST273 strains. Among seven *E. cloacae* isolates, two ST114 and one each of ST110, ST171, ST198, ST1252, and ST1303 strains were identified (Table 4).

### 2.3. Pulsed-Field Gel Electrophoresis (PFGE)

**Figure 1:**
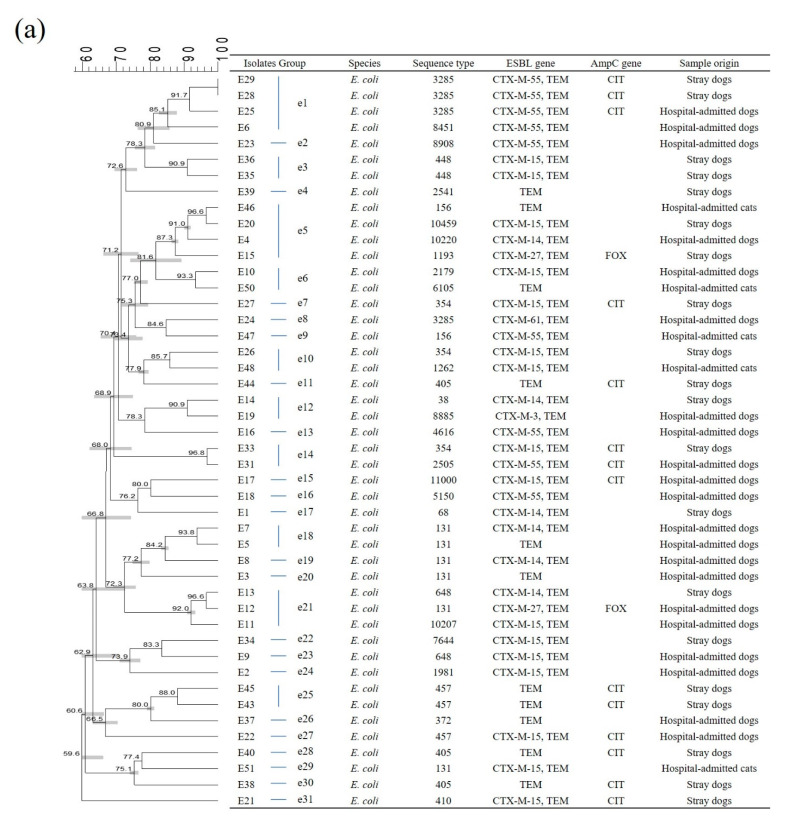

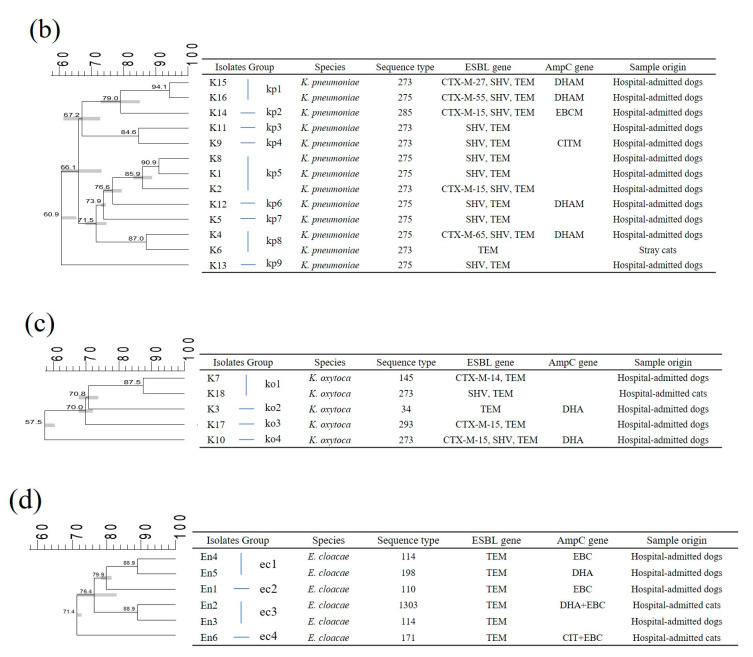
Dendrogram of pulsed-field gel electrophoresis (PFGE) patterns showing the genetic relatedness of ESBL-producing Enterobacterales isolates. (**a**) *Escherichia coli*, (**b**) *Klebsiella pneumoniae*, (**c**) *K. oxytoca*, and (**d**) *Enterobacter cloacae*.

PFGE analysis was only conducted for 46 *E. coli*, 13 *K. pneumoniae*, five *K. oxytoca*, and six *E. cloacae* isolates; no or few banding patterns were obtained for five *E. coli*, four *K. pneumoniae*, and one *E. cloacae* isolates. Using a >85% similarity cut-off, 31 pulsotypes in *E. coli* (e1 to e31), nine pulsotypes in *K. pneumoniae* (kp1 to kp9), four pulsotypes in *K. oxytoca* (ko1 and ko4), and four pulsotypes in *E. cloacae* (ec1 to ec4) were identified. Generally, all isolates demonstrated unique PFGE profiles (57–95% similarity), indicating genetic heterogeneity in ESBL- or AmpC-producing strains (Figure 1).

### 2.4. Genetic Relatedness

In *E. coli* PFGE analysis, the e1 group consisted of three ST3285 strains containing *bla*_CTX-M-55_*, bla*_TEM_, and *bla*_CIT_ from hospital-admitted and stray dogs and showed high similarity (>90%) (Figure 1a). Seven ST131 strains of *E. coli* isolates from five hospital-admitted dogs and two hospital-admitted cats were identified. PFGE results involved only five hospital-admitted dogs and one hospital-admitted cat, because the banding pattern for one hospital-admitted cat isolate was not defined. Two of them, which belonged to the e18 group, showed more than 93% similarity, compared with the remaining isolates in which low similarity was observed (<85%) (Figure 1a). e3 and e25 groups contained two ST448 strains with *bla*_CTX-M-55_ and *bla*_TEM_ and two ST457 strains with *bla*_TEM_ and *bla*_CIT_ from the same shelter and showed high similarity (>87%) (Figure 1a).

All *bla*_TEM_-positive *K*. *pneumoniae* isolates from hospital-admitted dogs harbored the *bla*_SHV_ gene and exhibited 60–95% similarity (Figure 1b). One *K. pneumoniae* ST273 strain from stray cats belonged to the kp8 group and showed 87% similarity with hospital-admitted dog isolates (Figure 1b). Two ST275 strains co-carrying *bla*_SHV_ and *bla*_TEM_, which belonged to the kp5 group, showed high similarity (>90%) (Figure 1b). The same pulsotype belonged to both ST273 and ST275, which were single-locus variants at the *tonB* allele; for example, kp1 and kp8. The remaining strains were independent of the groups obtained. Various STs and PFGE profiles were obtained for *K. oxytoca* and *E. cloacae* isolates, and genetic relatedness was not revealed for those strains (Figure 1c,d).

## 3. Discussion

This study presents the characteristics of 91 Enterobacterales isolates harboring ESC resistance genes, including *E. coli*, *Klebsiella* spp., *Serratia* spp., and *E. cloacae*, collected from South Korean companion animals between 2017 and 2019. All isolates harbored the *bla*_TEM_ gene and demonstrated unique PFGE profiles. Similarly, a study by Shin et al. revealed that all *E. coli* isolates from beef cattle harbored the *bla*_TEM_ gene [12]. Recent reports have identified CTX-M-type β-lactamases as the most widespread ESBL type, replacing classical TEM and SHV-type ESBLs [14]; however, TEM-type β-lactamases remained the most prevalent ESBL type identified in the current study. We observed varying predominant β-lactamase gene types in different Enterobacterales species, summarized as follows: *bla*_CTX-M_ and *bla*_CIT_ in *E. coli* isolates, *bla*_SHV_ and *bla*_DHA_ in *K. pneumoniae* isolates, and *bla*_EBC_ in *E. cloacae*. β-Lactamase gene distribution for each Enterobacterales species was similar to that described in a previous study with human samples [2]. These findings reveal that ESC resistance gene variants are not limited to certain hosts, emphasizing the need for coordinated control in both humans and animals.

In *E. coli* isolates in this study, *bla*_TEM_ and *bla*_CTX-M_ were most frequently detected, followed by *bla*_CIT_. Among *bla*_CTX-M_ positive isolates, *bla*_CTX-M-15_ was the most commonly detected gene followed by *bla*_CTX-M-55_ in both dogs and cats. A previous study investigating *E. coli* isolates from dogs reported that *bla*_CTX-M-15_, *bla*_CTX-M-14_, and *bla*_CIT_ were the most prevalent β-lactamase genes, whereas *bla*_CTX-M-55_ was rarely detected in South Korea [15]. However, *bla*_CTX-M-55_-carrying *E. coli* has become increasingly prevalent in dogs in South Korea [9]. The present study revealed that *bla*_CTX-M-55_ was predominantly detected rather than *bla*_CTX-M-14_ in *E. coli* from companion animals, which concurred with the results of the study by Hong et al. [9]. All five *E. coli* ST405 strains investigated in the current study harbored both *bla*_TEM_ and *bla*_CIT_ and were collected from stray dogs in the same shelter. The spread of *bla*_CIT_-carrying *E. coli* ST405 was described in a previous study, which suggested the possibility of direct transmission between humans and companion animals [9]. The spread of *E. coli* ST405 is usually described in humans harboring *bla*_CTX-M-15_ [16]. However, *E. coli* ST405 did not harbor *bla*_CTX-M-15_ in the current study. The increasing prevalence of *E. coli* ST131 carrying *bla*_CTX-M-15_ has been described in humans and animals [15,17]. Unexpectedly, only one *bla*_CTX-M-15_-carrying *E. coli* ST131 strain was detected from hospital-admitted cats in this study. From *E. coli* PFGE results, the two ST3285 strains showing the same PFGE pattern were both from the same shelter and isolated on the same date (Figure 1a). In this case, it could be the result of contaminated samples during sampling or transmission of a same clone between the two stray dogs in a shared place.

Among the seven *K. pneumoniae* and four *K. oxytoca* isolates harboring *bla*_CTX-M_ from hospital-admitted dogs in this study, the CTX-M-15 genotype accounted for a large proportion. A recent study also reported that *K. pneumoniae* isolates from companion animals producing CTX-M-15 either alone or in combination with DHA were frequently detected in South Korea [9]. The present MLST results revealed that ST275 and ST273 strains carrying both *bla*_SHV_ and *bla*_TEM_ were most commonly identified among *K. pneumoniae* isolates from hospital-admitted dogs. ST275 and ST273 are differentiated by one allele of the seven house-keeping genes, indicating that they are genetically close sequence types. Recently, the *bla*_SHV_- and *bla*_TEM_-co-carrying *K. pneumoniae* ST273 strain has emerged in human patients in Italy and is being disseminated, whereas ST273 and ST275 *Klebsiella* spp. isolates carrying both *bla*_SHV_ and *bla*_TEM_ have not yet been reported in South Korea [18,19]. Moreover, ST11, ST15, ST307, and ST392 strains have been globally identified as β-lactamase-producing *Klebsiella* spp. [9,20,21]. β-Lactamase-producing ST275 or ST273 strains among 

*Klebsiella* spp. isolates were newly discovered in South Korean companion animals in the current study.

All *E. cloacae* isolates in this study harbored *bla*_TEM_ and some carried *bla*_EBC_, whereas none carried *bla*_CTX-M_ and *bla*_SHV_. *E. cloacae* isolates from companion animals combined with more than two ESBL-type genes were described in Germany [22]. Meanwhile, *E. cloacae* isolates investigated in the present study harbored only one type of ESBL gene. In *Serratia* spp. isolates, only *bla*_TEM_ was detected, except one *S. marcescens* isolate that harbored both *bla*_TEM_ and *bla*_SHV_. An *S. marcescens* isolate carrying *bla*_TEM_ was previously identified in South Korea that caused urinary infections in humans [23]. However, in many countries including South Korea, the status of emerging AMR among *Serratia* spp. and *E. cloacae* in companion animals remains unknown. To our knowledge, this is the first report of ESC-resistant *Serratia* spp. and *E. cloacae* isolates from companion animals in South Korea.

In conclusion, we illustrated the presence and genetic heterogeneity of ESC-resistant Gram-negative bacteria in companion animals in South Korea, providing a potential reservoir of ESC-resistant bacteria and a transmission pathway. More organized surveillance is required to prevent and control the spread of ESC-resistant bacteria between companion animals and humans, in accordance with the “One Health” initiative.

## 4. Materials and Methods

### 4.1. Bacterial Characterization

Sampling, isolation, identification, antimicrobial susceptibility tests, and phenotypic characterization of Enterobacterales were previously studied [24,25]. In total, 91 Enterobacterales isolates carrying ESC resistance genes (51 *E. coli*, 17 *K. pneumoniae*, five *K. oxytoca*, four *S. marcescens*, seven *S. liquefaciens*, and seven *E. cloacae*) were collected from companion animals (56 hospital-admitted dogs, 23 stray dogs, 11 hospital-admitted cats, and one stray cat, Table 5). The antimicrobial resistance profile of the 91 bacterial isolates used in this study were summarized against cephalosporins (Table 5).

### 4.2. Characterization of β-Lactamase Genes

PCR amplification of entire *bla*_CTX-M_, *bla*_TEM_, and *bla*_SHV_ genes was performed as previously described [24,25]. For *bla*_CTX-M_-positive isolates, PCR and DNA sequencing were carried out for CTX-M-subtype detection. *bla*_CTX-M_ group-specific primers for five clusters (CTX-M-1, 2, 8, 9, and 25) were used following Kor-GLASS (Korea Global Antimicrobial Resistance Surveillance System) guidelines and previously published protocols [26,27]. DNA sequencing was performed by Intron Biotechnology (Seongnam, South Korea) and homologous sequences were searched against the GenBank database using the BLAST tool of the National Center for Biotechnology Information website (http://www.ncbi.nlm.nih.gov/BLAST (accessed on 15 December 2020)). In ESBL-positive strains, six groups of AmpC β-lactamases (MOX, CIT, DHA, ACC, EBC, and FOX) were screened by PCR amplification [28].

### 4.3. Multi-Locus Sequence Typing

Multi-locus sequence types (STs) were based on the allelic profile of seven housekeeping genes. For *E. coli*, *K. pneumoniae, K. oxytoca*, and *E. cloacae* isolates, MLST for each bacterial strain was carried out in reference to previous studies [29,30,31]. PCR was performed using primers for the following: *adk*, *fumC*, *gyrB*, *icd*, *purA*, *mdh*, and *recA* (*E. coli); gapA*, *infB*, *mdh*, *pgi*, *phoE*, *rpoB*, and *tonB* (*K. pneumoniae* and *K. oxytoca)*; *danA*, *fusA*, *gyrB*, *leuS*, *pyrG*, *rplB*, and *rpoB (E. cloacae).* Allelic profile and ST determinations were performed according to web-based MLST databases (https://pubmlst.org/databases/ (accessed on 15 December 2020) and https://bigsdb.pasteur.fr/klebsiella/klebsiella.html (accessed on 15 December 2020)).

### 4.4. Pulsed-Field Gel Electrophoresis

PFGE of *XbaI* (Takara Bio Inc., Shiga, Japan)-digested genomic DNA was carried out for *E. coli*, *K. pneumoniae*, *K. oxytoca*, and *E. cloacae* isolates according to the CDC PulseNet standardized procedure using the Chef Mapper system (Bio-Rad Laboratories, Hercules, CA, USA) [32]. PFGE analysis for *Serratia* spp. was ignored because different digested genomic DNA samples were employed for *Serratia* spp. Similarities between restriction fragment length polymorphisms were analyzed using GelCompar II software v. 6.5 (Applied Maths NV, St-Martens-Latem, Belgium) to produce a dendrogram. The unweighted-pair group method using average linkages (UPGMA) cluster analysis was conducted based on an 85% similarity cut-off with 0.5% optimization and 2.0% band tolerance.

## Figures and Tables

**Table 1:** Distribution of extended-spectrum β-lactamase (ESBL) genes among 91 Enterobacterales isolates from companion animals.

Organism	Hospital-Admitted Dogs (*n* = 56)			Stray Dogs (*n* = 23)			Hospital-Admitted Cats (*n* = 11)			Stray Cats (*n* = 1)			Total		
	*bla*_CTX-M_	*bla*_SHV_	*bla*_TEM_	*bla*_CTX-M_	*bla*_SHV_	*bla*_TEM_	*bla*_CTX-M_	*bla*_SHV_	*bla*_TEM_	*bla*_CTX-M_	*bla*_SHV_	*bla*_TEM_	*bla*_CTX-M_	*bla*_SHV_	*bla*_TEM_
*E. coli* (*n* = 51)	21	−	22	15	−	23	6	−	6	−	−	−	42 (82.4%)	−	51 (100%)
*K. pneumoniae* (*n* = 17)	7	15	15	−	−	−	1	1	1	1	−	1	9 (17.6%)	16 (94.1%)	17 (100%)
*K. oxytoca* (*n* = 5)	4	1	4	−	−	−	−	1	1	−	−	−	4 (80.0%)	2 (40.0%)	5 (100%)
*S. marcescens* (*n* = 4)	−	1	3	−	−	−	−	−	1	−	−	−	−	1 (45.0%)	4 (100%)
*S. liquefaciens* (*n* = 7)	−	−	7	−	−	−	−	−	−	−	−	−	−	−	7 (100%)
*E. cloacae* (*n* = 7)	−	−	5	−	−	−	−	−	2	−	−	−	−	−	7 (100%)

**Table 2:** *CTX-M* subtype detection of 55 *bla*_CTX-M_-positive Enterobacterales isolates.

Organism	Sample Origin	*bla*_CTX-M_ Subtype Group									Unidentified
		CTX-M-1					CTX-M-9				
		CTX-M-15	CTX-M-55	CTX-M-3	CTX-M-61	Total	CTX-M-14	CTX-M-27	CTX-M-65	Total	
*E. coli* (*n* = 42)	Hospital-admitted dogs (*n* = 21)	7	6	1	1	15	3	1	−	4	2
	Stray dogs (*n* = 15)	8	3	−	−	11	3	1	−	4	0
	Hospital-admitted cats (*n* = 6)	2	2	−	−	4	−	−	−	0	2
*K. pneumoniae* (*n* = 9)	Hospital-admitted dogs (*n* = 7)	4	1	−	−	5	−	1	1	2	0
	Hospital-admitted cats (*n* = 1)	−	−	1	−	1	−	−	−	0	0
	Stray cats (*n* = 1)	−	−	−	−	0	−	−	−	0	1
*K. oxytoca* (*n* = 4)	Hospital-admitted dogs (*n* = 4)	2	−	−	−	2	1	−	−	1	1
Total		23 (41.8%)	12 (21.8%)	2 (3.6%)	1 (1.8%)	38 (69.1%)	7 (12.7%)	3 (5.5%)	1 (1.8%)	11 (20.0%)	6 (10.9%)

**Table 3:** Distribution of AmpC β-lactamase genes among ESBL-producing Enterobacterales isolates.

Organism	Sample Origin	AmpC β-Lactamases Gene								
		MOX	CIT	DHA	ACC	EBC	FOX	EBC+CIT	EBC+DHA	Total
*E. coli*	Hospital-admitted dogs (*n* = 22)	−	5	−	−	−	−	−	−	5
	Stray dogs (*n* = 23)	−	13	−	−	−	1	−	−	14
*K. pneumoniae*	Hospital-admitted dogs (*n* = 15)	−	1	4	−	1	−	−	−	6
	Hospital-admitted cats (*n* = 1)	−	−	1	−	−	−	−	−	1
*K. oxytoca*	Hospital-admitted dogs (*n* = 4)	−	−	2	−	−	−	−	−	2
*E. cloacae*	Hospital-admitted dogs (*n* = 5)	−	−	1	−	2	−	−	−	3
	Hospital-admitted cats (*n* = 2)	−	−	−	−	−	−	1	1	2
Total		−	19	8	−	3	1	1	1	33

**Table 4:** Multi-locus sequence typing (MLST) results based on the spread of β-lactamase resistance genes.

Organism	*bla*_CTX-M_ Cluster	ESC Resistance Gene				No. Isolation			
		ESBLs			AmpCs	ST Type			
		*bla*_CTX-M_	*bla*_SHV_	*bla*_TEM_		Hospital-Admitted Dogs	Stray Dogs	Hospital-Admitted Cats	Stray Cats
*E. coli*	CTX-M-1	CTX-M-15	−	+	−	648 (*n* = 1)	354 (*n* = 1)	131 (*n* = 1)	−
						1981 (*n* = 1)	448 (*n* = 2)	1262 (*n* = 1)	−
						2179 (*n* = 1)	7644 (*n* = 1)	−	−
						10,207 (*n* = 1)	10,459 (*n* = 1)	−	−
			−	+	CIT	457 (*n* = 1)	354 (*n* = 2)	−	−
						5667 (*n* = 1)	410 (*n* = 1)	−	−
						11,000 (*n* = 1)	−	−	−
		CTX-M-55	−	+	−	4616 (*n* = 1)	−	131 (*n* = 1)	−
						5150 (*n* = 1)	−	156 (*n* = 1)	−
						8451 (*n* = 1)	−	−	−
						8908 (*n* = 1)	−	−	−
			−	+	CIT	2505 (*n* = 1)	410 (*n* = 1)	−	−
						3285 (*n* = 1)	3285 (*n* = 2)	−	−
	CTX-M-9	CTX-M-61	−	+	−	3285 (*n* = 1)	−	−	−
		CTX-M-3	−	+	−	8885 (*n* = 1)	−	−	−
		CTX-M-14	−	+	−	10,220 (*n* = 1)	38 (*n* = 1)	−	−
						131 (*n* = 2)	68 (*n* = 1)	−	−
						−	648 (*n* = 1)	−	−
		CTX-M-27	−	+	−	131 (*n* = 1)	−	−	−
		CTX-M-27	−	+	FOX	−	1193 (*n* = 1)	−	−
						−	−	−	−
	Unidentified	−	−	+	−	131 (*n* = 2)	−	156 (*n* = 1)	−
						−	−	6105 (*n* = 1)	−
	Negative	−	−	+	−	372 (*n* = 1)	2541 (*n* = 1)	−	−
		−	−	+	CIT	−	405 (*n* = 5)	−	−
						−	457 (*n* = 2)	−	−
Total						22	23	6	0
*K. pneumoniae*	CTX-M-1	CTX-M-15	+	+	−	273 (*n* = 2)	−	−	−
						275 (*n* = 1)	−	−	−
			+	+	EBC	285 (*n* = 1)	−	−	−
		CTX-M-55	+	+	DHA	275 (*n* = 1)	−	−	−
		CTX-M-3	+	+	DHA	−	−	273 (*n* = 1)	−
	CTX-M-9	CTX-M-27	+	+	DHA	273 (*n* = 1)	−	−	−
		CTX-M-65	+	+	DHA	275 (*n* = 1)	−	−	−
	Unidentified	−	-	+	−	−	−		273 (*n* = 1)
	Negative	−	+	+	−	273 (*n* = 2)	−	−	−
						275 (*n* = 4)	−	−	−
		−	+	+	CIT	273 (*n* = 1)	−	−	−
		−	+	+	DHA	275 (*n* = 1)	−	−	−
Total						15	0	1	1
*K. oxytoca*	CTX-M-1	CTX-M-15	−	+	−	293 (*n* = 1)	−	−	−
			+	+	DHA	273 (*n* = 1)	−	−	−
	CTX-M-9	CTX-M-14	−	+	−	145 (*n* = 1)	−	−	−
	Unidentified	−	−	+	DHA	34 (*n* = 1)	−	−	−
	Negative	−	+	+	−	−	−	273 (*n* = 1)	−
Total						4	0	1	0
*S. liquefaciens*	Negative	−	−	+	−	^a^ ND (*n* = 7)	−	−	−
*S. marcescens*	Negative	−	−	+	−	ND (*n* = 2)	−	ND (*n* = 1)	−
			+	+	−	ND (*n* = 1)	−	−	−
Total						10	0	1	0
*E. cloacae*	Negative	−	−	+	DHA	198 (*n* = 1)	−	−	−
		−	−	+	EBC	114 (*n* = 1)	−	−	−
						110 (*n* = 1)	−	−	−
		−	−	+	CIT+EBC	−	−	171 (*n* = 1)	−
		−	−	+	DHA+EBC	−	−	1303 (*n* = 1)	−
		−	−	+	−	1252 (*n* = 1)	−	−	−
						114 (*n* = 1)	−	−	−
Total						5	0	2	0

^a^ ND, non-defined.

**Table 5:** The source and antimicrobial resistance characteristics of 91 bacterial isolates used in this study.

Organism	Origin	Antimicrobial Resistant Rate against Cephalosporins (%)					Reference
		Cephalexin	Cefoxitin	Ceftiofur	Ceftriaxone	Cephalothin	
*E. coli* (*n* = 51)	Hospital-admitted dogs (*n* = 22)	95.5	27.3	95.5	95.5	100	[25]
	Stray dogs (*n* = 23)	100	47.8	100	100	100	
	Hospital-admitted cats (*n* = 6)	100	16.7	100	100	100	[24]
*K. pneumonia* (*n* = 17)	Hospital-admitted dogs (*n* = 15)	53.3	60.0	33.3	40	53.3	[25]
	Hospital-admitted cats (*n* = 1)	100	100	100	100	100	[24]
	Stray cat (*n* = 1)	100	100	100	100	100	
*K. oxytoca* (*n* = 5)	Hospital-admitted dogs (*n* = 4)	75.0	50.0	100	100	100	[25]
	Hospital-admitted cat (*n* = 1)	100	100	0	0	100	[24]
*S. marcescens* (*n* = 4)	Hospital-admitted dogs (*n* = 3)	100	100	0	0	100	[25]
	Hospital-admitted cat (*n* = 1)	100	100	0	0	100	[24]
*S. liquefaciens* (*n* = 7)	Hospital-admitted dogs (*n* = 7)	0	14.3	28.6	0	100	[25]
*E. cloacae* (*n* = 7)	Hospital-admitted dogs (*n* = 5)	100	100	60.0	80.0	100	[25]
	Hospital-admitted cats (*n* = 2)	100	100	100	100	100	[24]

## Data Availability

The data presented in this study are available on request from the corresponding author.

## Abbreviations

AmpC: AmpC β-lactamase; AMR, antimicrobial resistance; ESBL, extended-spectrum β-lactamase; ESC, extended-spectrum cephalosporin; MLST, multi-locus sequence typing; PFGE, pulsed-field gel electrophoresis; ST, sequence type.
